# Mutant CAG Repeats Effectively Targeted by RNA Interference in SCA7 Cells

**DOI:** 10.3390/genes7120132

**Published:** 2016-12-17

**Authors:** Agnieszka Fiszer, Joanna P. Wroblewska, Bartosz M. Nowak, Wlodzimierz J. Krzyzosiak

**Affiliations:** Department of Molecular Biomedicine, Institute of Bioorganic Chemistry, Polish Academy of Sciences, Noskowskiego 12/14 Str., 61-704 Poznan, Poland; wroblewska.jp@gmail.com (J.P.W.); poz.bartosznowak@gmail.com (B.M.N.)

**Keywords:** spinocerebellar ataxia type 7, siRNA, CAG repeats, polyglutamine diseases, allele-selective silencing

## Abstract

Spinocerebellar ataxia type 7 (SCA7) is a human neurodegenerative polyglutamine (polyQ) disease caused by a CAG repeat expansion in the open reading frame of the *ATXN7* gene. The allele-selective silencing of mutant transcripts using a repeat-targeting strategy has previously been used for several polyQ diseases. Herein, we demonstrate that the selective targeting of a repeat tract in a mutant *ATXN7* transcript by RNA interference is a feasible approach and results in an efficient decrease of mutant ataxin-7 protein in patient-derived cells. Oligonucleotides (ONs) containing specific base substitutions cause the downregulation of the *ATXN7* mutant allele together with the upregulation of its normal allele. The A2 ON shows high allele selectivity at a broad range of concentrations and also restores *UCHL1* expression, which is downregulated in SCA7.

## 1. Introduction

Polyglutamine (polyQ) diseases are a group of neurological disorders caused by an open reading frame (ORF)-located CAG repeat expansion in specific genes, and they include Huntington’s disease (HD), dentatorubral-pallidoluysian atrophy (DRPLA), spinal bulbar muscular atrophy (SBMA), and spinocerebellar ataxia (SCA) types 1, 2, 3, 6, 7 and 17 [1]. The CAG repeat tract in the *ATXN7* gene mutated in SCA7 reaches 37–300 repeats (usually 40–60 CAGs). Ataxin-7 functions in the cytoplasm in the regulation of cytoskeletal dynamics [2], whereas in the nucleus it is a key component of the SPT3-TAF31-GCN5L acetyltransferase (STAGA) complex, which is involved in transcriptional regulation [3]. In SCA7, the impaired expression of microRNA (miR)-124 due to STAGA dysfunction was found to be responsible for neurodegeneration in specific tissues [4]. Also, caspase-mediated cleavage of mutant protein was found to be a critical event in SCA7 disease pathogenesis [5]. The expansion of the polyQ tract in ataxin-7 leads to its accumulation in nuclear inclusions and to the selective degeneration of neurons in the cerebellum (loss of the Purkinje cells is a characteristic feature) and photoreceptors in the retina. Various pathways impaired in neurons in SCA7 are identified [6,7]. As a result of degeneration, a phenotype characterized by ataxia and visual impairment is observed in SCA7 patients [8,9].

There are few described examples of *ATXN7* silencing with RNA interference (RNAi) tools, and for allele-selective downregulation of the mutant allele, only a single nucleotide polymorphism (SNP)-targeting strategy has been extensively tested [10,11,12]. In one approach, short hairpin RNA (shRNAs) and primary microRNA (pri-miR)–based reagents (shmiRs) were developed and tested in a cellular model expressing *ATXN7* exogenes [13]. The targeting of a common SNP variant, which is also linked to an *ATXN7* mutation, resulted in the high discrimination of silencing. In another study, synthetic small interfering RNA (siRNAs) were tested in SCA7 fibroblasts [14]. *ATXN7* silencing was demonstrated at the transcript level, and there was a lack of selectivity in a broad range of siRNA concentrations. A non-allele-selective approach using shmiR was tested in a SCA7 mouse model [15,16]. The expression of both *ATXN7* alleles was downregulated using RNAi specifically in the retina or Purkinje cells, and widespread beneficial effects were observed.

CAG repeat–targeting RNAi reagents containing base substitutions were successfully tested for HD, SCA3 and DRPLA, and various types of reagents were developed for this strategy, including short duplexes, self-duplexing guide-only siRNAs, shRNA, and chemically modified single-stranded siRNAs [17,18,19,20,21,22,23,24]. These reagents formed mismatches with their target and triggered translational inhibition, rather than transcript degradation [25]. In this study we used an SCA7 model not yet explored for this strategy, and we briefly report on mutant *ATXN7* silencing by selected oligonucleotides (ONs). Our results are promising from the perspective of RNAi-based therapy for SCA7 patients.

## 2. Materials and Methods

### 2.1. Cell Culture

Fibroblasts from SCA7 patient (GM03561, 8/62 CAG in *ATXN7* gene) and control fibroblasts (GM00024, GM07492 and GM07525—marked as F1, F2 and F3 in figures, respectively) were obtained from the Coriell Cell Repositories (Camden, NJ, USA) and grown in minimal essential medium (Sigma-Aldrich, St. Louis, MO, USA) supplemented with 10% or 15% fetal bovine serum (FBS) (Sigma-Aldrich), antibiotics (Sigma-Aldrich), GlutaMAX (ThermoFisher Scientific, Waltham, MA, USA) and non-essential amino acids (Sigma-Aldrich). 

### 2.2. Oligonucleotides and Transfection

**Figure 1:**
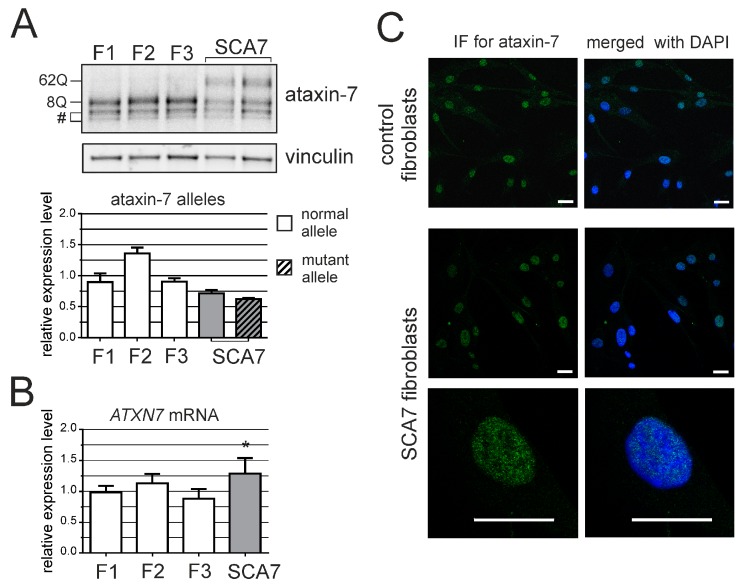
*ATXN7* expression in human fibroblasts. (**A**) Western blot analysis of ataxin-7 levels in control (F1, F2 and F3) and spinocerebellar ataxia type 7 (SCA7) fibroblasts. Representative blot is shown and a graph presenting quantitation based on analyses from three separate protein isolations. In the case where the expression level of individual alleles was analyzed separately, clear bars represent normal allele and hatched bars represent mutant allele; (**B**) Quantitative Reverse transcription polymerase chain reaction (qRT-PCR) analysis of total *ATXN7* mRNA levels in control and SCA7 fibroblasts; (**C**) Representative images of anti-ataxin-7 immunofluorescence (IF) in fibroblast cell lines (control: GM07492 and SCA7). Scale bar = 25 μm. 4′,6-diamidino-2-phenylindol (DAPI) staining of the nuclei is in blue.

RNA ON and chemically modified ONs were synthesized by FutureSynthesis (Poznan, Poland) or IDT (Coralville, IA, USA). The sequences of oligonucleotides used in this study are presented in Figure 1. Cell transfections were performed using Lipofectamine 2000 transfection reagent (Life Technologies) according to the manufacturer’s instructions. The transfection efficiency was monitored using 20 nM BlockIT fluorescent siRNA (Life Technologies). Due to the rapid growth of the SCA7 cell line, the medium was changed to complete medium after 4 h from transfection to complete medium containing 5% FBS.

### 2.3. Reverse Transcription Polymerase Chain Reaction and Quantitative Reverse Transcription Polymerase Chain Reaction

Total RNA was isolated from fibroblast cells using TRIzol reagent (Sigma-Aldrich) and a Direct-zol kit (Zymo Research, Irvine, CA, USA) according to the manufacturer’s instructions. The RNA concentration was measured using a DeNovix spectrophotometer (Wilmington, DE, USA). A total of 500 ng of RNA was reverse transcribed at 55 °C using Superscript III (Life Technologies) and random hexamer primers (Promega, Madison, WI, USA). Complementary DNA (cDNA) was used for quantitative polymerase chain reaction (qPCR) using LightCycler 480 SYBR Green I Master (Roche, Basel, Switzerland) with denaturation at 95 °C for 10 min followed by 45 cycles of denaturation at 95 °C for 10 s, annealing at 60 °C for 15 s and elongation at 72 °C at 20 s, with *ATXN7*, *UCHL1* or *GAPDH*-specific primers (sequences are listed in Table 1) on the Light Cycler 480 II (Roche). Data pre-processing and normalization were performed using LightCycler 480 SW 1.5.1 software (Roche). For semi-quantitative PCR, GoTaq polymerase (Promega) was used, and reaction products were separated on 1.5% agarose gels in 0.5× TBE buffer and stained with ethidium bromide. 

### 2.4. Western Blot

First 25 μg of total protein isolated with PB buffer (60 mM Tris-base, 2% SDS, 10% sucrose, 2 mM PMSF) was run on NuPAGE 3%–8% Tris-Acetate gels (Thermo Fisher Scientific) in Tris-Acetate SDS Running Buffer (Life Technologies) at 4 °C. The immunoreaction was performed using the following antibodies: anti-ataxin-7 (NBP1-42657, Novus Biologicals, Littleton, CO, USA), anti-vinculin (4650, Cell Signaling Technology, Danvers, MA, USA), and anti-rabbit horseradish peroxidase (HRP)-conjugate (Jackson ImmunoResearch, West Grove, PA, USA,) and detected using WesternBright Quantum HRP Substrate (Advansta, Menlo Park, CA, USA). The protein bands were scanned directly from the membrane using a camera and were quantified using the Gel-Pro Analyzer (Media Cybernetics, Rockville, MD, USA).

### 2.5. Immunofluorescence

Immunofluorescence labeling was performed with an anti-ataxin-7 antibody (PA1-749, ThermoFisher Scientific) at a 1:100 dilution and secondary anti-rabbit antibody conjugated with Alexa Fluor 488 (Jackson ImmunoResearch) at a 1:500 dilution. For nuclei staining SlowFade Gold with DAPI (ThermoFisher Scientific ) was used.

### 2.6. Statistical Analysis

The statistical significance of the silencing was assessed using a one-sample *t*-test, with an arbitrary value of 1 assigned to cells treated with control siRNA. Selected data were compared using an unpaired *t*-test with Welch’s correction to assess the allele-selectivity of silencing (normal vs. mutant allele silencing). Two-tailed *p*-values below 0.05 were considered significant. All experiments with statistical analyses were repeated at least three times.

## 3. Results

We used a human fibroblast cell line derived from a SCA7 patient as a model for testing selected RNAi-based ONs. This required the optimization of Western blot analysis for ataxin-7, as no results have yet been published that present endogenous mutant ataxin-7 downregulation. Normal and mutant ataxin-7 were separated and clearly detected (Figure 1A). We analyzed *ATXN7* expression in three control lines of fibroblasts and the SCA7 line. ATXN7 mRNA and protein levels (total of both alleles) were upregulated in the mutant cell line by ≈25% compared with the average *ATXN7* expression level in control cell lines (Figure 1A,B). *ATXN7* expression upregulation in SCA7 cells was previously observed [4]. Preferential nuclear localization of ataxin-7 was observed in control and SCA7 fibroblasts (Figure 1C).

**Figure 2:**
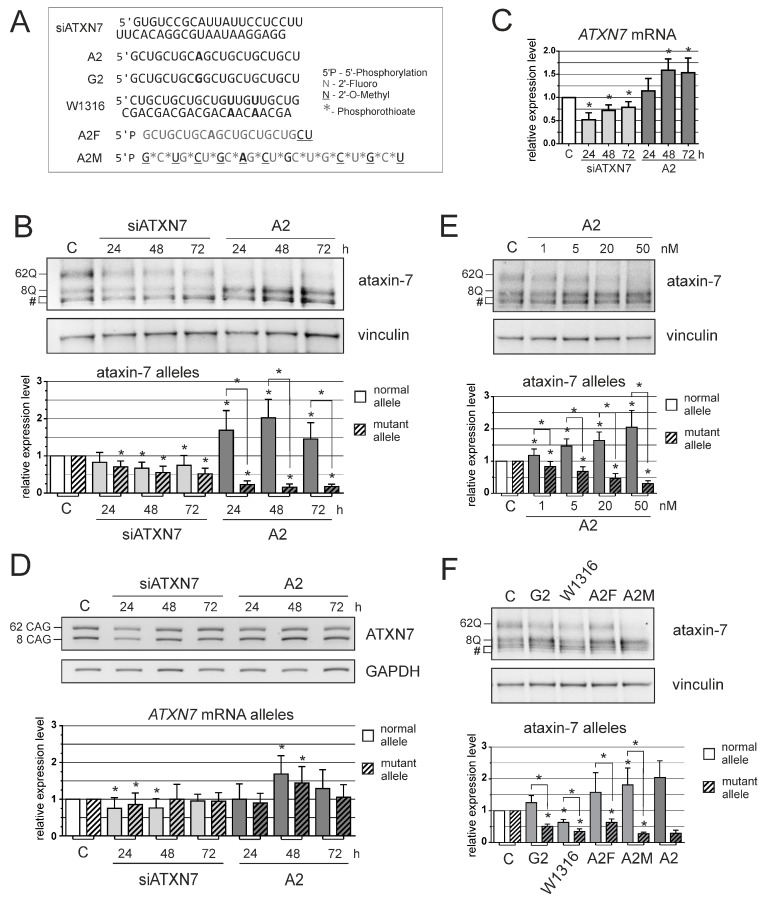
Efficiency and selectivity of small interfering RNA (siRNAs) assessed in human SCA7 fibroblasts. (**A**) The nucleotide sequences and chemical modifications of the tested oligonucleotides (ONs). Base substitutions resulting in mismatch formation with the target sequence are marked in bold; (**B**) Western blot analysis of ataxin-7 levels in SCA7 fibroblasts at 24, 48 or 72 h after transfection with 100 nM of the indicated siRNA; (**C**) qRT-PCR analysis of total *ATXN7* mRNA levels in SCA7 fibroblasts for the same experiment as in (**B**); (**D**) Reverse transcription polymerase chain reaction (RT-PCR) analysis of normal and mutant *ATXN7* allele expression level for the same experiment as in (**B**); (**E**) Western blot analysis of ataxin-7 levels in SCA7 fibroblasts at 48 h after transfection with 1, 5, 20 or 50 nM siRNA A2; (**F**) Western blot analysis of ataxin-7 levels in SCA7 fibroblasts at 48 h after transfection with 50 nM indicated siRNAs. Results for A2 were also included as a reference. #—unspecific bands; C—control line, SCA7 fibroblasts transfected with BlockIT siRNA. In the case where the expression level of individual alleles was analyzed separately, clear bars represent normal allele and hatched bars represent mutant allele. The error bars represent standard deviations. The *p*-value is indicated with an asterisk (* *p* < 0.05).

Next, we compared the efficiency and selectivity in the downregulation of the ataxin-7 protein for *ATXN7* sequence-specific siRNA (siATXN7) and selected a CAG repeat–targeting ON: self-duplexing (sd)-siRNA A2 [21]. We investigated the activity of siATXN7 and A2 (Figure 2A) at 24, 48 and 72 h post-transfection at 100 nM. The expression of *ATXN7* was analyzed using Western blot and Reverse transcription polymerase chain reaction (RT-PCR). Sequence-specific siRNA caused lowering of both ataxin-7 alleles level up to 50% of control levels (Figure 2B). Sd-siRNA A2 showed high efficiency in mutant protein downregulation (to ≈20% of the control level), and normal protein upregulation was also observed (to ~200% of the control level). Protein downregulation was also analyzed at 96 h post-transfection, and a substantial silencing effect was still observed (data not shown). Both quantitative polymerase chain reaction (qPCR) and semi-quantitative PCR were used for the quantitation of total *ATXN7* mRNA, and separate analyses were performed for the normal and mutant alleles (Figure 2C,D). siATXN7 caused a decrease in the transcript level to ≈50% of the control levels 24 h after transfection, and this effect was reduced at the subsequent time-points analyzed. The total level of *ATXN7* mRNA was upregulated after A2 transfection to ≈150% of control levels at the 48 and 72 h time-points (Figure 2C). A separate analysis of *ATXN7* alleles by semi-quantitative RT-PCR revealed a similar general trend of changes in *ATXN7* mRNA level, namely downregulation of *ATXN7* transcript by siATXN7 at the first time points analyzed, a lack of decrease of the *ATXN7* mRNA level after treatment with A2 at all time points analyzed and upregulation of the *ATXN7* transcript by A2 at 48 h post-transfection (Figure 2D).

We then investigated the silencing of *ATXN7* expression at the protein level by A2 at lower concentrations of 1, 5, 20 and 50 nM (Figure 2E). Significant mutant ataxin-7 silencing was already observed at a 1 nM concentration, but downregulation to less than 50% of the control level required 20 nM. Significant upregulation of normal allele expression was observed starting from a 5 nM concentration. It is noteworthy that A2 caused a significant allele selectivity of *ATXN7* silencing for the full spectrum of concentrations investigated.

We also analyzed other ONs, which are similar to A2 in containing base substitutions in a repeated sequence and a chemically modified version of A2. We tested sd-siRNA G2 [21], W1316 duplex [18] and A2F and A2M [26] (Figure 2A). A2F possesses a chemical modification pattern containing mainly 2′-fluoro (2′F) as well as two 2′-*O*-methylo (2′*O*Me) nucleotides from the 3′ end, while A2M contains 2′F, 2′*O*Me and phosphorothioate modifications. These ONs were transfected to SCA7 fibroblasts at a 50 nM concentration and showed diverse profiles of efficiency and selectivity in *ATXN7* silencing (Figure 2F). W1316 was the least selective one, while A2M caused similar effects to non-modified A2, i.e., the downregulation of mutant ataxin-7 to ~25% of the control level and upregulation of the normal allele to ~150% of the control level.

**Figure 3:**
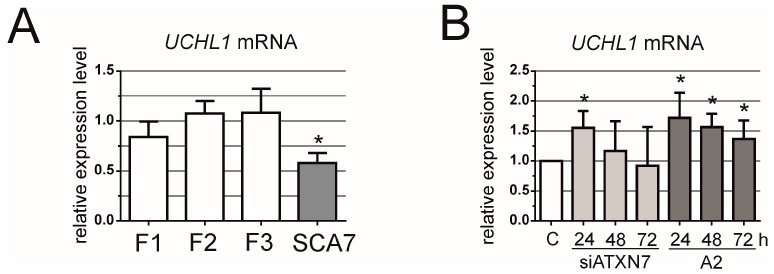
Downstream effects of *ATXN7* silencing. (**A**) qRT-PCR analysis of *UCHL1* mRNA levels in control (F1, F2 and F3) and SCA7 fibroblasts; (**B**) qRT-PCR analysis of total *ATXN7* mRNA levels in SCA7 fibroblasts for siATXN7 and A2 at 24, 48 or 72 h after transfection with 100 nM siRNAs. C—control line, SCA7 fibroblasts transfected with BlockIT siRNA. The error bars represent standard deviations. The *p*-value is indicated with an asterisk (* *p* < 0.05).

Finally, we investigated the downstream effects caused by mutant ataxin-7 downregulation. We selected the *UCHL1* gene, encoding the de-ubiquitinating enzyme, which was shown earlier to be silenced together with *ATXN7* with a decreased expression level [14]. The *UCHL1* mRNA level was decreased in SCA7 fibroblasts compared with the control cell lines (Figure 3A). Both ONs tested, siA7 and A2, reversed this effect and caused the upregulation of *UCHL1* expression. The effect was more pronounced for A2 as it occurred at all time-points of post-transfection analyzed, and the upregulation reached 175% of the control level (Figure 3B).

## 4. Discussion

Standard RNAi technology uses siRNAs fully complementary to specific sequences in mRNA to induce AGO2-mediated transcript cleavage and degradation. We used a modified approach characterized by targeting the CAG repeat region in mRNA with siRNAs containing base mismatches with the target. By testing this atypical RNAi strategy, we demonstrated that A2 siRNA shows high allele selectivity for silencing the mutant gene causing SCA7. A2 siRNA causes an efficient decrease of mutant ataxin-7 (by ≈75%) and, importantly, simultaneously upregulates normal ataxin-7, which can also be regarded as the desired effect for therapeutic purposes. Mutant *ATXN7* silencing occurs at the protein level, without inducing considerable mutant mRNA degradation. The possible explanation of observed effects is the upregulation of both *ATXN7* alleles at the mRNA level and preferential translational inhibition of the mutant allele. A similar upregulation caused by other CAG repeat–targeting ONs was observed for normal huntingtin protein [18]. Some aspects of the mechanism, by which these so-called miRNA-like siRNAs function, was previously investigated [21,25] proving the AGO2-dependent activity and suggesting the cooperative action of multiple RNA-induced silencing complexes (RISCs) bound to the expanded CAG repeat tract.

RNAi is continuously regarded as a very potent therapeutic tool despite further advances in developing antisense oligonucleotides [27] and the more recently invented clustered regularly interspaced short palindromic repeats (CRISPR)-based tools [28,29]. All these therapeutic strategies are aimed at the elimination of the cause of the disease, i.e., mutant gene expression. This is a very promising approach for polyQ diseases, which still remain incurable. The reduction of expression of the mutant *ATXN7* transgene by ≈50% in the inducible SCA7 mouse model resulted in the reduction of ataxin-7 aggregation and the reversal of behavioral abnormalities [30], which is a proof-of-concept for RNAi-based strategies.

In our studies, we focus on the allele-selective approach, which targets mutant allele expression with high preference. Preserving normal ataxin-7 activity is desirable considering its important function. The use of non-allele-selective RNAi-based reagents directed to Purkinje cells was shown to be tolerated and beneficial in an animal model of SCA7 but only a ≈25% decrease in endogenous protein level was reported [16].

Allele-selective SNP-targeting strategies require the presence of SNP variants discriminating between the normal and mutant alleles. The selectivity of silencing in the CAG repeat–targeting strategy is dependent on the repeat tract length, but it is more universal and is also applicable for other polyQ diseases. 

## Figures and Tables

**Table 1:** Sequences of PCR primers. Oligonucleotides (ONs) used for repeat tract amplification are marked with star.

Name	Sequence 5′-3′
GAPDH F	GAAGGTGAAGGTCGGAGTC
GAPDH R	GAAGATGGTGATGGGATTTC
ATXN7 F	AGGTGTTCTTAGCGCATCCT
ATXN7 R	AGTGTGCCATCCATTTTCGG
ATXN7* F	ACCCTCCAAAGAAAAGGAGCG
ATXN7* R	AGCATCACTTCAGGACTGGG
UCHL1 F	GGAAGGCCAATGTCGGGTAG
UCHL1 R	GCAGGGTGTCCTCTGAACTG
